# Optimization of the Formula and Processing Factors for Gluten-Free Rice Bread with Tamarind Gum

**DOI:** 10.3390/foods9020145

**Published:** 2020-02-01

**Authors:** Ye-Eun Hong, Meera Kweon

**Affiliations:** 1 Department of Food Science and Nutrition, Pusan National University, Busan 46241, Korea; yea1244@naver.com; 2 Kimchi Institute, Pusan National University, Busan 46241, Korea

**Keywords:** gluten-free, rice bread, tamarind gum, factorial design, optimization, formula, processing factor

## Abstract

The formula and processing parameters for gluten-free rice bread were optimized using a factorial design, including a center point. Gum concentration (GC), water amount (WA), mixing time (MT), and fermentation time (FT) were selected as factors, and two levels were used for each factor: 1 and 2% for GC; 80 and 100 g for WA; 5 and 10 min for MT; and 30 and 60 min for FT. The WA and FT were identified as the most significant factors in determining the quality of gluten-free rice bread with tamarind gum. Thus, the optimized formula and processing conditions for maximizing bread volume and minimizing bread firmness were 1% gum, 100 g water, 5 min MT, and 60 min FT. The addition of an anti-staling enzyme reduced the increase in bread firmness and the enthalpy of starch retrogradation, suggesting its potential for successful application in commercially manufactured rice bread with tamarind gum.

## 1. Introduction

In Korea, with increasing personal income and the westernization of dietary patterns, the consumption of rice is decreasing, while the use of wheat is increasing [1]. Wheat contains glutenin and gliadin, which are unique protein components that form the viscoelastic gluten essential for bread-making. However, omega gliadin, present in wheat, is a major component that causes celiac disease, which is an autoimmune disease caused by ingesting gluten, and its main symptoms include chronic diarrhea, vomiting, and abdominal distension in young infants [2]. Patients with celiac disease are advised to consume gluten-free foods from grains that do not contain gliadin. Rice is one such grain commonly used for gluten-free products. Thus, the development of gluten-free foods using rice flour is considered to be a suitable strategy to increase rice consumption [3].

Considerable research on the development of gluten-free foods using rice has been actively conducted worldwide [4,5,6], and various gluten-free products are being sold commercially. Baked goods are popular, and many studies have been performed to develop gluten-free rice bread. However, the improvement of the pseudo-viscoelasticity imparted by rice flour dough is still needed to develop a gluten-free rice product whose volume and texture are similar to those of bread made of wheat flour. Many types of grains, protein sources, and gums have been studied to improve the quality of gluten-free bread [7,8].

Several gums, such as xanthan gum, guar gum, pectin, carrageenan, and hydroxyl methylcellulose (HPMC), have been studied [9,10,11,12,13], and, recently, tamarind gum has been reported for its suitability in the manufacturing of rice bread [14]. Tamarind gum is a xyloglucan and can improve bread-making by increasing the stability of the dough and loaf volume [15]. The majority of studies on gluten-free products have investigated the ingredients and formulation, with less focus on the processing conditions. Therefore, in-depth research on the formulation and processing conditions of gluten-free rice bread, with the added tamarind gum, is needed for its successful application.

In this study, a factorial design was used to identify and optimize significant factors in the formulation and processing conditions of gluten-free rice bread with the addition of tamarind gum. Gum concentration (GC), water amount (WA), mixing time (MT), and fermentation time (FT) were the four factors selected, and two levels were set for each factor. Quality characteristics of the gluten-free rice bread were evaluated by measuring the pH of batter before and after fermentation, moisture, volume, and crumb texture of the bread. The effect of an anti-staling enzyme on gluten-free rice bread at the optimal formulation and processing conditions, selected through the experiment, was analyzed for quality changes during cold storage by measuring moisture, texture, and the retrogradation enthalpy of the rice bread by differential scanning calorimetry (DSC).

## 2. Materials and Methods

### 2.1. Materials

The rice flour used (Nongsim, Seoul, Korea) was a wet mill product purchased from a local market. Tamarind gum was obtained from a manufacturing company (Socius Ingredients LLC, Evanston, IL, USA), and sugar (CJ CheilJedang Corp, Seoul, Korea), soy milk powder (Benesoy, Rock City, IL, USA), canola oil (Beksul, Seoul, Korea), salt (Haepyo, Seoul, Korea), and dry yeast (Lesaffre, Marcqen–Baroeul, France) were purchased from a local market. The anti-staling enzyme Novamyl 10,000 BG, used in the storage experiments, was obtained from a manufacturing company (Novozymes, Bagsvaerd, Denmark).

### 2.2. Preparation of Rice Bread

The Design Expert (Minneapolis, MN, USA) program was used for the factorial design to identify and optimize the significant factors of the formulation and processing conditions. The four factors—concentration of gum, amount of water, mixing time, and fermentation time—were selected, with each factor consisting of two levels: gum concentration at 1 and 2%; water at 80 and 100 g; mixing time at 5 and 10 min; and fermentation time at 30 and 60 min. The basic ingredients and formulation used were 100 g of rice flour, 5 g of canola oil, 7 g of sugar, 3 g of soy milk powder, 2 g of salt, and 3 g of yeast, according to the combined method described by Hera et al. [16] and Jang et al. [14]. The rice bread was prepared as per the method described by Jang [17].

Pre-weighed dry ingredients, including rice flour and tamarind gum, were tumbled in a jar with a lid for uniform blending and transferred to a mixer bowl (Pin Mixer, National Mfg. Co., Lincoln, NE, USA). Oil (5 g) and water (amount set as per the experimental design shown in Table 1) were added and mixed for a set time. Batter (190 g) was placed in a baking pan (a non-stick mini loaf pan, 14.6 cm × 8.3 cm × 5.7 cm; Chicago Metallic, Lake Zurich, IL, USA). The baking pan containing the batter was placed in a fermenter (Samjung, Gyeonggi, Korea) for a set time at 30 °C and 85% relative humidity. Batter appearance, before and after fermentation, was photographed using a digital camera (Canon, Tokyo, Japan). After fermentation, 10 g of the batter was removed for pH measurement, and the remaining batter was baked in an oven (Phantom M301 Combi, Samjung, Gyeonggi, Korea) at 215 °C for 20 min. After cooling at room temperature (25–30 °C) for 20 min, the bread was taken out of the baking tray. It was further cooled for 40 min before quality analysis. Bread baking was repeated twice for each condition. 

### 2.3. Analysis of Batter Property of Rice Bread

To measure the specific gravity of the batter, 5 g of the batter was placed in a 50 mL beaker, and its volume was marked. After removing the batter, water was poured up to the mark, and the volume and weight of the water were measured. The specific gravity of the batter was calculated.

To measure the pH before and after fermentation of the batter, 5 g of batter and 50 g of distilled water were mixed in a blender (SFM-7700JJH, Sinil, Korea) for 20 s to ensure uniform dispersion, and the batter suspension was transferred to a beaker. The pH of the suspension was measured using a pH meter (Mettler Toledo SevenEasy pH meter S20, Columbus, OH, USA).

### 2.4. Evaluation of Rice Bread Quality

Bread weight, height, and volume were measured according to the method described by Jang et al. [14]. To measure bread volume, millet seeds were used in the seed replacement method. The density (g/mL) of millet, used for converting weight into volume, was determined by filling a 1.5 L plastic container with millet. A loaf of bread was placed in the container filled with millet, and the weight of millet that was pushed out of the container (g) was measured. The volume of the bread (mL) was calculated based on the calculated density.

According to the AACC Approved Method 74–09.01 [18] and that described by Jang et al. [14], bread firmness was measured using a Texture Analyzer (Brookfield CT3, Middleboro, MA, USA). The measurement conditions were set as follows: mode, measure force in compression; pre-test speed, 2.0 mm/s; test speed, 2.0 mm/s; post-test speed, 5.0 mm/s; probe, a 3.6 cm diameter cylinder; and penetration distance, 15 mm. One loaf of bread was cut from the centre into two pieces with a thickness of 2 cm; its firmness was measured by compressing the centre of the cross-section of the bread.

The moisture content of the bread samples was measured using the AACC Approved Method 44–15.02 [18]. Approximately 3 g of pre-weighed breadcrumbs in an aluminium container was placed in a hot-air drying oven (FO 600-M, Jeio Tech, Daejeon, Korea), dried at 130 °C for 1 h, and cooled at room temperature in a desiccator for 30 min. The moisture content of the bread was calculated from the weight reduced after drying.

### 2.5. Evaluation of the Effect of an Anti-staling Enzyme on the Staling of Rice Bread during Storage

To evaluate the effect of an anti-staling enzyme on bread staling during storage, bread was prepared as per the formulation and processing conditions were selected as the most desirable in the optimization experiment conducted in this study. An anti-staling enzyme, Novamyl 10,000 BG, was added to the bread formulation at 0.005, 0.01, and 0.015%, and mixed well with the dry ingredients used to prepare the rice bread as per the abovementioned process. Control bread, for comparison, was made without adding the enzyme. One loaf of bread was cut from the centre into three pieces with a thickness of 2 cm. One piece was used as the fresh sample (0 days); the other two pieces were sealed in aluminium foil packaging to avoid moisture loss during storage and placed in a refrigerator at 4 °C. Measurements of firmness, moisture content, and enthalpy of starch retrogradation of bread by DSC during storage were performed on 0, 1, and 4 days of storage after baking.

The temperature and enthalpy of starch retrogradation of bread were measured by DSC (DSC 6000, Perkin–Elmer Co., Waltham, MA, USA). Breadcrumbs and water were mixed well at a ratio of 1:1 (*w*/*w*). Then, 40 mg of the mixture was sealed in a stainless steel pan and heated at 10 °C/min of heating rate from 10 °C to 140 °C using a differential scanning calorimeter. The temperature and enthalpy of starch retrogradation of bread were analyzed using Pyris Software (ver. 11, Perkin–Elmer Co.).

### 2.6. Statistical Analysis

All results were evaluated by two or more repeated experiments and analyzed using the Design Expert program to identify significant factors, as well as to optimize the formulation and processing conditions. The SPSS statistical program (ver. 22.0, IBM Corp., Armonk, NY, USA) was used for the ANOVA and the Duncan’s multiple range test at a significance threshold of *p* < 0.05.

## 3. Results and Discussion

### 3.1. Characteristics of the Batter of Rice Bread

**Figure 1:**
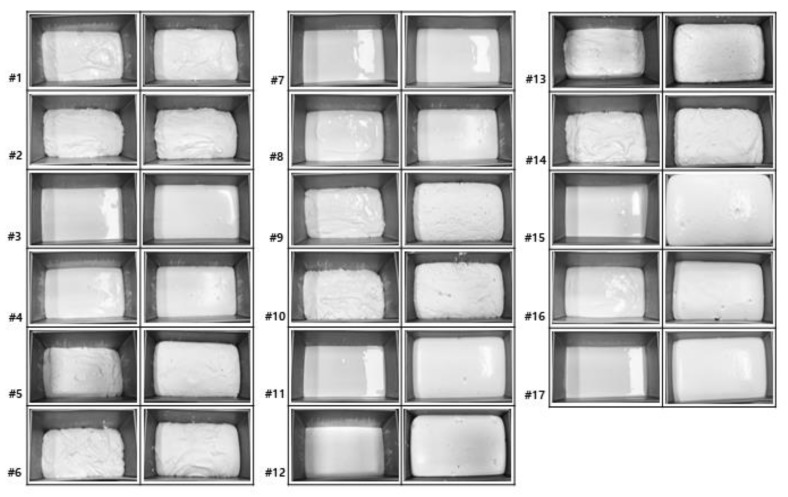
Appearance of batter prepared as per the designed formula and processing conditions before (**left**) and after (**right**) fermentation.

The appearance of the batter, prepared according to the experimental design, is presented in Figure 1. With the high WA in the formulation, the batter was thin. Within the same WA in the formulation, the batter with the high GC was relatively thicker. With the increasing WA and FT, the expanded volume of the batter after fermentation increased. The most significant increase in batter volume after fermentation was observed in batters #11, 12, 15, and 16, which were formulated with 100 g of water and fermented for 60 min, and #17, the center point with 90 g of water for 45 min of fermentation. Increasing the FT could break the dough structure by weakening the hydrocolloid network in which starch granules or flour particles are embedded, resulting in less resistance to strain [19]. The extent of volume expansion between these batter samples was not significantly different by varying the MT. However, when the GC was less, the batter looked thin, which might have resulted in easy expansion. A lower batter consistency is preferred for good performance during leavening [20]. Thus, the appearance of the batter might be linked to the bread volume.

The pH of the rice bread batter, before and after fermentation, is shown in Table 2. After fermentation, the pH of all the samples decreased, which was considered to be the result of acidic substances, such as lactic acid and acetic acid, that were produced during fermentation [21]. The pH change during fermentation was 0.3–0.7. The batter samples (#1–8), fermented for 30 min, showed a pH of 5.7–5.9 after the fermentation, resulting in a pH change of 0.3–0.5, whereas the samples (#9–16), fermented for 60 min, showed a pH of 5.4–5.6 after fermentation, resulting in a pH change of 0.5–0.7. As a result, it is suggested that the fermentation time affected the batter pH, whereas the GC, WA, and MT did not show any significant effects on batter pH. Jang et al. [14] reported that changes in batter pH during fermentation were different between various gums used at 4%. However, no significant difference was observed at the 1–2% concentration of tamarind gum that was used in this study in order to follow the U.S. Food and Drug Administration (FDA) regulation.

The specific gravity of all of the batter samples was 1.1 g/cc, which indicated no significant difference in aeration during mixing due to a negligible difference (1 and 2%) in the viscosity of the gum. But, Jang [17] observed differences in the specific gravity of the batter depending on the type of gum used in the gluten-free rice bread. When the hydroxypropyl methylcellulose (HPMC) concentration was increased from 1 to 4% in the formulation of rice bread, the specific viscosity of the batter was significantly increased because of the increased thickness that caused less aeration during mixing.

### 3.2. Quality Characteristics of Rice Bread

**Figure 2:**
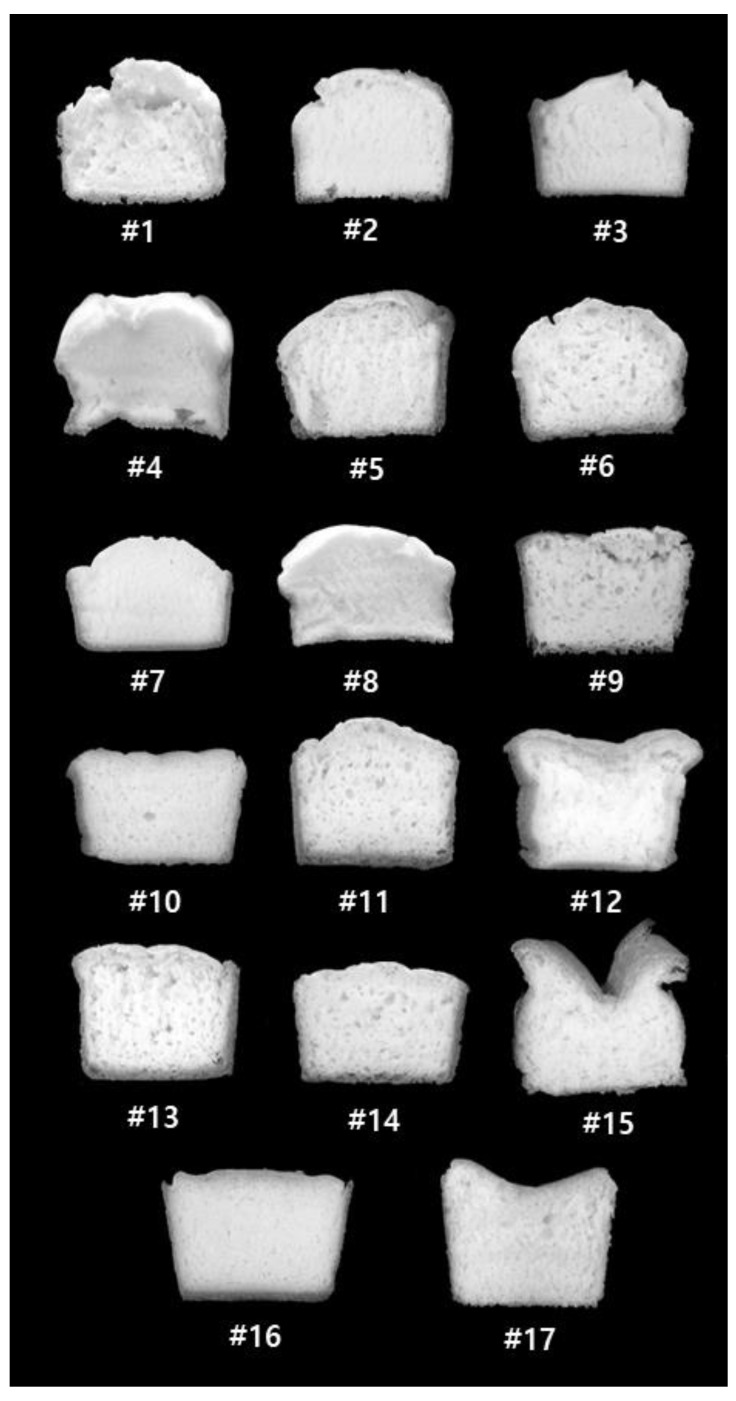
Cross-sections of gluten-free rice bread tested by a full factorial design.

The cross-section of the rice bread is shown in Figure 2. The #9–16 bread samples with 60 min of fermentation exhibited a relatively higher volume than the #1–8 bread samples with 30 min of fermentation, reflecting the effect of FT on the bread volume. The impact of the WA was more pronounced at a longer FT, and the #11, 12, 15, and 16 bread samples exhibited relatively large cross-sectional volumes of bread. Among the bread samples, #11 (1% gum, 100 g of water, 5 min MT, and 60 min FT) was selected as the most desirable bread that was similar to commercial bread, based on crumb pore size and symmetrical shape.

The weight of the bread was affected significantly by the high WA with 60 min FT but not with 30 min FT. The weight of the former bread per loaf was 144.1–145.6 g, whereas that of the latter was 150.3–153.2 g (Table 2). When the WA was high and the FT long, the batter could expand quickly during the baking process, resulting in an increase in volume and moisture loss during baking [5, 16]. The volume of rice bread was also affected significantly by the FT with high WA. At 60 min of fermentation, the volume of bread was 338.3–380.0 mL, whereas, at 30 min, the volume of bread was 270.6–293.5 mL (Table 2). When the WA was low, the effect of the FT on bread volume was not significant.

The moisture content and firmness of the breadcrumbs are shown in Table 2. The moisture content of the breadcrumbs correlated with the amount of water in the formulation. Regardless of the FT, the water content of the crumbs was 44.4–46.3% when a small amount of water was added to the formulation and 49.3–51.3% when a large amount of water was added to the formulation. The firmness of the breadcrumbs showed a significant relationship with the moisture content of the breadcrumbs in the bread samples prepared with the same FT. When the FT is long (60 min) and the WA is large, the bread volume is large and the firmness of the bread crumb is significantly low [5,16,22]. Overall, the quality of bread was affected by the WA and FT, which were the significant factors, but was not affected by the GC and MT.

### 3.3. Significant Factors Affecting the Quality Characteristics of Rice Bread

**Figure 3:**
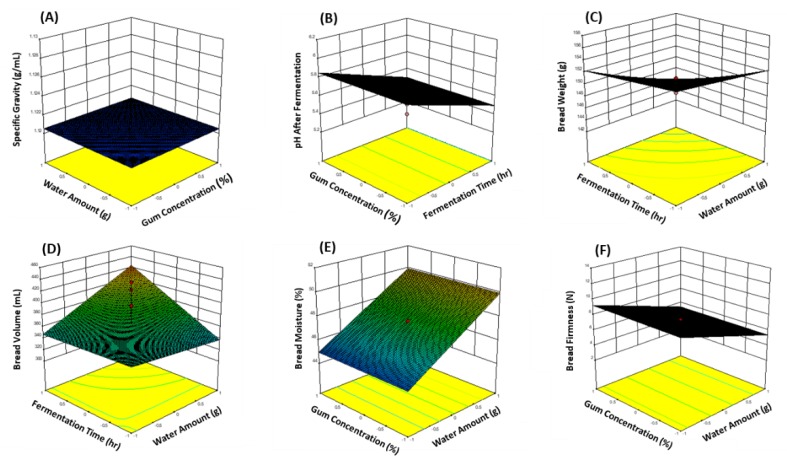
Three-dimensional plots on the characteristics of batter (**A**,**B**) and bread (**C**–**F**) affected by significant factors of the formula and processing conditions in the preparation of gluten-free rice bread with tamarind gum.

The results of the statistical analysis of the batter characteristics and bread quality characteristics of gluten-free rice bread prepared based on the full factorial design of a factor analysis method are shown in Table 3. The three-dimensional plots of the six characteristics of gluten-free rice batter and bread with tamarind gum are presented as a function of the two independent variables among GC, WA, MT, and FT in response to their significance in Figure 3. 

With regard to the batter characteristics, no significant factors affected the specific gravity (Figure 3A) or pH before fermentation; however, FT was a significant factor that was negatively correlated with the pH after fermentation. It was predicted that the pH of the batter decreased after fermentation with the increase in the FT (Figure 3B).

With regard to the quality characteristics of bread, the WA and FT were the significant factors negatively correlated with the weight of bread. The weight of the bread decreased as the WA and FT increased (Figure 3C). However, FT was positively correlated with bread volume, confirming that the FT needs to be controlled to increase the volume. Interaction of WA and FT also had a positive effect on bread volume. Thus, the bread volume increased as the WA and FT increased (Figure 3D). The moisture content of breadcrumbs was positively correlated with the WA, whereas the firmness of the breadcrumbs was negatively correlated with the WA (Figure 3E,F). These results reflected the importance of the WA for bread quality. Hera et al. [16] and Mancebo et al. [5] reported the effect of water content on gluten-free bread quality; the bread specific volume increased and the crumb texture improved by increasing dough hydration, which was similar to our results.

### 3.4. Effect of an Anti-staling Enzyme on the Quality of Rice Bread during Storage

**Figure 4:**
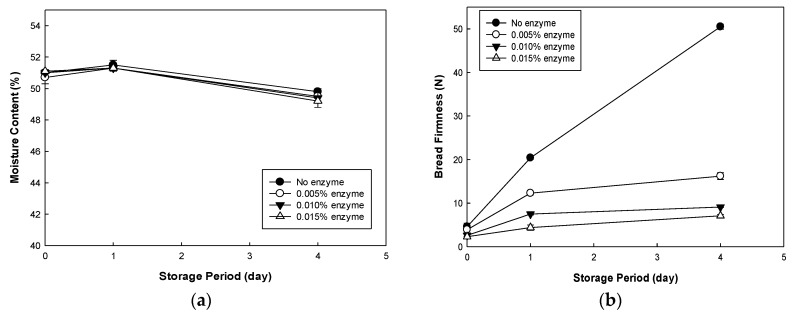
Changes in the moisture content (**a**) and firmness (**b**) of bread with anti-staling enzyme at different concentrations during storage at 4 °C.

The moisture content and firmness of bread after adding an anti-staling enzyme during storage at 4 °C and its retrogradation tendency measured by DSC are shown in Figure 4 and Table 4. The control rice bread without the added enzyme showed a significant increase in firmness during storage compared to the bread with the added enzyme. The results of the control bread were consistent with the storage test of rice bread with tamarind gum reported by Jang et al. [14]. Gluten-free rice bread has a higher starch percentage than wheat bread, and numerous attempts to retard staling have been reported [23,24].

The firmness of the control rice bread before refrigerated storage was 4.6 N and that of the bread with the added enzyme was slightly less (2.3–3.9 N) (Figure 4). With the increasing amount of enzyme, the firmness of the bread decreased. With the increasing storage period, the firmness increased. The difference between the control and the enzyme addition group was more significant on day four. The firmness of bread after refrigerated storage increased from 4.6 to 50.5 N for the control group, from 3.9 to 16.2 N for the 0.005% enzyme group, from 2.6 to 9.1 N for the 0.010% enzyme group, and from 2.3 to 9.1 N for the 0.015% enzyme group. As the amount of enzyme increased, the extent of the increase in firmness during storage significantly decreased (*p* < 0.05), demonstrating the effective anti-staling impact of the enzyme. The reaction of maltogenic amylase is known to hydrolyze starch in the batter during mixing and baking and reduce the size of amylose and amylopectin. Shorter amylose and amylopectin cannot rearrange easily, resulting in retardation of starch retrogradation [25].

The moisture content of the rice bread during refrigerated storage was 51.1–49.2% (Figure 4), which was the most favorable condition for starch retrogradation [26]. During storage, it showed a slight change on the fourth day for all samples, indicating the excellent packaging of the bread during storage. The bread with the enzyme showed less of an increase in firmness than the control bread, demonstrating the anti-staling effect of the enzyme during storage.

The enthalpy of starch retrogradation of bread with and without the enzyme during storage, measured by DSC, was compared (Table 4). As the storage period increased, the peak of the control bread in the DSC thermogram increased significantly; however, that of the bread with the enzyme did not increase dramatically. Moreover, as the amount of enzyme increased, the increase in peak size during storage decreased. The enthalpy of starch retrogradation of bread increased with an increase in the storage period. The control bread showed the highest increase in enthalpy, whereas that with the enzyme showed a less significant increase in enthalpy. The extent of the increase in enthalpy decreased as the amount of added enzyme increased. The retrogradation enthalpy on the fourth day of storage was 1.43, 1.11, 1.02, and 0.73 J/g for the control bread and 0.005, 0.010, and 0.015% enzyme added bread, respectively. α-Amylase hydrolyzes starch while increasing sugars, which can be fermented by the yeast. The enzyme reaction increases the bread volume, improves the color and flavor of the bread crust, and extends the shelf life by retarding staling [27]. In this study, the addition of the enzyme inhibited the increase in firmness of gluten-free rice bread during storage, indicating the excellent anti-staling effect. This finding suggests its potential for successful application in commercially manufactured rice bread.

## 4. Conclusions

A factorial analysis was used to identify and optimize significant factors in the formulation and processing conditions of rice bread with tamarind gum. Four factors—gum concentration, water amount, mixing time, and fermentation time—were selected and two levels for each factor were set, making a total of 17 conditions, including the center point. Although WA and FT significantly affected the quality of gluten-free rice bread, GC and MT did not. Overall, the WA and FT were identified as the significant factors in the production of gluten-free rice bread. Therefore, proper control over WA and FT can maximize the bread volume and minimize the firmness of the bread. Among the various experimental conditions, 1% tamarind gum, 100 g of water, 5 min of mixing, and 60 min of fermentation were determined to be the optimal conditions. The addition of the anti-staling enzyme was effective in retarding the increase in retrogradation enthalpy and bread firmness. Using an optimized formula and processing factors for gluten-free rice bread with the combined addition of tamarind gum and an anti-staling enzyme can be applied successfully in commercially manufactured gluten-free rice bread.

## Figures and Tables

**Table 1:** Experimental conditions tested by a full factorial design for preparing gluten-free rice bread.

Std Order	Run Order	Gum (%)	Water (g)	Mixing Time (min)	Fermentation Time (min)
1	12	1	80	5	30
2	14	2	80	5	30
3	9	1	100	5	30
4	16	2	100	5	30
5	3	1	80	10	30
6	5	2	80	10	30
7	10	1	100	10	30
8	17	2	100	10	30
9	13	1	80	5	60
10	7	2	80	5	60
11	11	1	100	5	60
12	2	2	100	5	60
13	1	1	80	10	60
14	6	2	80	10	60
15	4	1	100	10	60
16	15	2	100	10	60
17	8	1.5	90	7.5	45

**Table 2:** pH of batter before and after fermentation and gluten-free rice bread characteristics tested by a full factorial design.

Std Order	Batter		Bread			
	pH before Fermentation	pH after Fermentation	Weight (g)	Volume (mL)	Moisture Content (%)	Firmness (N)
1	6.2 ± 0.1 ^a,^*	5.9 ± 0.1 ^a^	153.8 ± 0.2 ^b,c^	289.8 ± 11.5 ^b^	46.2 ± 0.0 ^a^	9.8 ± 1.1 ^e,f^
2	6.3 ± 0.2 ^a^	5.9 ± 0.2 ^a^	154.1 ± 0.5 ^b,c^	300.1 ± 0.9 ^b^	45.3 ± 0.3 ^a^	8.9 ± 0.1 ^d,e,f^
3	6.2 ± 0.0 ^a^	5.9 ± 0.1 ^a^	150.7 ± 0.2 ^b,c^	273.2 ± 5.0 ^a,b^	50.0 ± 0.6 ^c^	7.0 ± 1.2 ^b,c,d,e^
4	6.3 ± 0.2 ^a^	5.9 ± 0.2 ^a^	153.2 ± 0.7 ^b,c^	370.1 ± 6.0 ^d,e^	49.9 ± 0.5 ^c^	6.8 ± 1.2 ^b,c,d,e^
5	6.0 ± 0.1 ^a^	5.7 ± 0.1 ^a^	152.9 ± 0.9 ^b,c^	295.2 ± 5.0 ^b^	45.6 ± 0.7 ^a^	8.5 ± 0.9 ^c,d,e^
6	6.0 ± 0.1 ^a^	5.7 ± 0.2 ^a^	154.6 ± 1.1 ^c^	276.2 ± 14.1 ^a,b^	44.4 ± 0.3 ^a^	8.8 ± 1.0 ^d,e,f^
7	6.2 ± 0.0 ^a^	5.8 ± 0.0 ^a^	152.3 ± 0.8 ^b,c^	258.3 ± 4.5 ^a^	51.3 ± 0.1 ^c^	7.0 ± 0.6 ^b,c,d,e^
8	6.3 ± 0.3 ^a^	5.8 ± 0.2 ^a^	152.2 ± 1.4 ^b,c^	274.3 ± 8.8 ^a,b^	50.6 ± 0.8 ^c^	7.0 ± 0.2 ^b,c,d,e^
9	6.2 ± 0.0 ^a^	5.5 ± 0.0 ^a^	151.1 ± 0.3 ^b,c^	282.7 ± 3.5 ^a,b^	46.3 ± 0.3 ^a^	7.9 ± 1.0 ^c,d,e^
10	6.1 ± 0.2 ^a^	5.5 ± 0.2 ^a^	152.0 ± 0.1 ^b,c^	270.6 ± 6.2 ^a,b^	44.4 ± 0.2 ^a^	9.9 ± 1.1 ^e,f^
11	6.2 ± 0.1 ^a^	5.6 ± 0.0 ^a^	145.3 ± 1.1 ^a^	338.3 ± 3.8 ^c^	50.6 ± 0.7 ^c^	4.0 ± 0.1 ^a,b^
12	6.1 ± 0.2 ^a^	5.5 ± 0.1 ^a^	145.2 ± 0.6 ^a^	360.0 ± 12.4 ^c,d,e^	50.4 ± 1.0 ^c^	5.5 ± 0.4 ^a,b,c^
13	6.0 ± 0.1 ^a^	5.5 ± 0.1 ^a^	153.2 ± 4.1 ^b,c^	293.5 ± 12.8 ^b^	45.2 ± 1.3 ^a^	12.0 ± 2.0 ^f^
14	6.0 ± 0.1 ^a^	5.4 ± 0.2 ^a^	150.3 ± 0.6 ^b^	275.3 ± 0.8 ^a,b^	44.4 ± 0.2 ^a^	8.5 ± 0.9 ^c,d,e^
15	6.1 ± 0.1 ^a^	5.4 ± 0.2 ^a^	144.1 ± 0.2 ^a^	349.1 ± 8.0 ^c,d^	49.3 ± 0.3 ^b,c^	3.1 ± 0.3 ^a^
16	6.3 ± 0.3 ^a^	5.6 ± 0.2 ^a^	145.6 ± 1.0 ^a^	380.0 ± 10.9 ^e^	50.6 ± 0.3 ^c^	4.0 ± 1.2 ^a,b^
17	6.0 ± 0.2 ^a^	5.5 ± 0.2 ^a^	149.9 ± 0.5 ^b^	365.6 ± 18.8 ^c,d,e^	47.0 ± 2.4^a,b^	5.8 ± 1.1 ^a,b,c,d^

* Results are expressed as the mean ± standard deviation. Values with the same letter within the same column are not significantly different (*p* < 0.05), according to Duncan’s multiple range test.

**Table 3:** Significant factors for the batter and bread characteristics of gluten-free rice bread tested by a full factorial design.

Sample	Response	Significant Factor	*p*-Value	Significance
Batter	Specific gravity	-	-	-
	pH before fermentation	-	-	-
	pH after fermentation	Fermentation time (FT)	0.0002	Negative
Bread	Weight	Fermentation time	<0.0001	Negative
		Water amount (WA)	<0.0001	Positive
		WA × FT	0.0024	Positive
	Volume	Fermentation time	0.0207	Positive
		WA × FT	0.0097	Positive
	Moisture content	Water amount	0.0002	Positive
	Firmness	Water amount	<0.0001	Negative

**Table 4:** Thermal characteristics of gluten-free rice bread prepared with #11 (1% gum, 100 g of water, a 5 min mixing time, and a 60 min fermentation time) during storage at 4 °C.

Enzyme Concentration (%)	Storage Time (day)	Tonset (°C)	Tpeak (°C)	Tend (°C)	Heat of Transition (ΔQ, J/g)
0	0	40.1 ± 0.1 ^a,^*	48.1 ± 0.1 ^a^	66.9 ± 1.1 ^c,d,e,f^	0.35 ± 0.0 ^a^
	1	40.0 ± 0.0 ^a^	53.4 ± 0.1 ^b^	64.8 ± 0.2 ^b,c,d,e^	0.82 ± 0.0 ^c^
	4	40.8 ± 0.9 ^a^	52.8 ± 0.4 ^b^	65.1 ± 0.6 ^b,c,d,e^	1.43 ± 0.1 ^e^
0.005	0	40.0 ± 0.0 ^a^	48.0 ± 0.1 ^a^	67.8 ± 0.9 ^e,f^	0.28 ± 0.0 ^a^
	1	40.0 ± 0.0 ^a^	52.8 ± 0.6 ^b^	64.6 ± 0.0 ^b,c,d^	0.60 ± 0.0 ^b^
	4	40.7 ± 0.3 ^a^	52.8 ± 0.6 ^b^	64.6 ± 0.7 ^b,c,d^	1.11 ± 0.1 ^d^
0.010	0	40.18 ± 0.1 ^a^	48.7 ± 0.1 ^a^	68.4 ± 0.3 ^f^	0.37 ± 0.0 ^a^
	1	40.0 ± 0.0 ^a^	52.7 ± 0.2 ^b^	61.0 ± 0.8 ^a^	0.40 ± 0.1 ^a^
	4	40.47 ± 0.1 ^a^	51.4 ± 2.1 ^b^	64.6 ± 0.6 ^b,c,d^	1.02 ± 0.1 ^d^
0.015	0	40.0 ± 0.0 ^a^	48.5 ± 1.2 ^a^	67.6 ± 2.4 ^d,e,f^	0.25 ± 0.0 ^a^
	1	40.0 ± 0.0 ^a^	53.0 ± 0.5 ^b^	63.8 ± 0.7 ^a,b,c^	0.36 ± 0.1 ^a^
	4	40.79 ± 0.7 ^a^	52.7 ± 0.8 ^b^	63.5 ± 0.0 ^a,b^	0.73 ± 0.0 ^b,c^

* Results are expressed as the mean ± standard deviation. Values with the same letter within the same column are not significantly different (*p* < 0.05), according to Duncan’s multiple range test.
